# Phage-induced protection against lethal bacterial reinfection

**DOI:** 10.1073/pnas.2423286122

**Published:** 2025-05-30

**Authors:** Yikun Xing, Haroldo J. Hernandez Santos, Ling Qiu, Samantha R. Ritter, Jacob J. Zulk, Rachel Lahowetz, Kathryn A. Patras, Austen L. Terwilliger, Anthony W. Maresso

**Affiliations:** ^a^TAILOR Labs, Baylor College of Medicine, Houston, TX 77030; ^b^Department of Molecular Virology and Microbiology, Baylor College of Medicine, Houston, TX 77030; ^c^Alkek Center for Metagenomics and Microbiome Research, Baylor College of Medicine, Houston, TX 77030

**Keywords:** ExPEC, immunity, phage therapy, sepsis

## Abstract

Bacteriophages, or phages, are viruses that target and infect bacteria. Due to a worldwide rise in antimicrobial resistance (AMR), phages have been proposed as a promising alternative to antibiotics for the treatment of resistant bacterial infections. Up to this point in history, phage use in preclinical animal studies, clinical trials, and emergency-use compassionate care cases has centered around the original observation from 1915 showing phage as lytic agent, and thus a treatment that kills bacteria. Here, we describe an activity associated with phage therapy that extends beyond lytic activity that results in long-term protection against reinfection. This activity is potent, providing almost complete protection against a second lethal infection for animals treated with phage therapy. The activity also reduced infection burden an astounding billion-fold over the control. Reinfection protection requires phage lytic killing of its target bacterium but is independent of additional phage therapy. The effect is not driven by phage alone, lingering phage resistors, or a sublethal inoculum. In vitro phage-lysed bacteria provide partial protection, suggesting a combination of phage-induced lytic activity and immune stimulation by phage treatment is responsible for the effect. These observations imply certain phages may induce host adaptive responses following the lysis of the infecting bacteria. This work suggests phage therapy may contain a dual-action effect, an initial treatment efficacy followed by a long-term protection against reoccurring infection, a therapeutic-vaccination mechanism of action.

## Significance

In 2021, antimicrobial-resistant bacteria were responsible for 1.14 million deaths and associated with 4.71 million deaths globally. Patients who experience sepsis often face a higher risk of reinfections and hospital readmissions. To combat this crisis, bacteriophages—viruses that infect and kill bacteria—are regaining interest as a potential solution. Here, we show that mice infected with extraintestinal pathogenic *Escherichia coli* and treated with phage HP3 not only recover from the initial infection but also gain protection against a secondary challenge with the same bacterial strain. The protective effect is dependent on the bacteriolytic action of the phage. These findings shift phages from being solely therapeutic antimicrobials to dual-action immunotherapeutics capable of both clearing and preventing bacterial infections.

Once considered the silver bullet for bacterial infections in the 1940s, antibiotics are now a catalyst of a looming health crisis. In 2019, there were 13.7 million infection-related deaths worldwide, and 7.7 million were associated with bacterial pathogens (1). Specifically, bacterial antimicrobial resistance (AMR) caused 1.27 million deaths and was linked to other 4.95 million deaths globally (2). Along with high mortality rates, AMR bacterial infections are a financial burden, costing $4.6 billion in the United States in 2017 alone (3). In their 2023 report, the United Nations Environment Programme warned that by the year 2050, AMR could be responsible for up to 10 million deaths per year and called for multisectoral approaches to combat superbugs (4). Recently, others have calculated potential deaths at 39 million individuals by the year 2050 (5).

Bacteremia (bacteria in blood) and other bacterial infections can cause sepsis, a life-threatening condition where the host’s immune response is dysregulated, leading to tissue damage and organ dysfunction (6). In 2017, sepsis ranked as the most costly medical condition in the United States, with expenses totaling $38.2 billion (7). Globally, there were approximately 48.9 million reported cases and 11 million sepsis-related deaths in 2017 (8). In 2021, the number of sepsis-related patient stays in the United States reached 2.5 million, with a 16.5% in-hospital mortality rate, and the hospital costs amounted to $52.1 billion, though the statistics should be considered in light of the COVID-19 pandemic’s impact (9). A systematic review and meta-analysis revealed that 48.7% of sepsis cases with organ dysfunction in the ICU were hospital-acquired, with a 52.3% mortality rate (10). Moreover, retrospective cohort studies show sepsis survivors have increased risk of readmission due to infections within 90 d (11, 12) to a year (13) posthospitalization. Needless to say that bloodstream infections are of great concern because of their high AMR and potential lethality.

The Gram-negative bacterial pathogen *Escherichia coli* plays a significant role in these statistics. Per a recent systematic review on ExPEC bacteremia, the estimated incidence rate in high-income countries was 48 per 100,000 person-years (14). *E. coli* alone accounted for 950,000 deaths across several infectious syndromes such as peritoneal and intra-abdominal infections (290,000), bloodstream infections (242,000), and urinary tract infections (UTIs) (120,000) (1). Although *Staphylococcus aureus* was the deadliest pathogen worldwide (1), *E. coli* was responsible for the most deaths associated with bacterial AMR (2). Extra Intestinal Pathogenic *E. coli*, or ExPEC is the name given to *E. coli* that causes a diverse array of invasive infections (UTI, meningitis, etc.), has a mixture of different virulence factors and AMR, and can be transmitted from animals to humans and humans to humans (15). The high mortality and high healthcare costs of ExPEC infections represent a public health concern that requires both infection prevention and treatment measures. Despite research efforts to develop an ExPEC vaccine, none has been FDA-approved (16, 17). Due to AMR, bacterial-killing viruses known as bacteriophages have been touted as a promising solution to combat AMR, in particular to counter ExPEC (18, 19).

Bacteriophages, or phages, are viruses that exhibit strain-specific killing of bacteria, making them effective alternatives to antibiotics. There are more phages than stars in the knowable universe, indicating a limitless supply of antibacterial diversity (20). Tailed phages attach to their target bacteria through highly specific interactions between their tail fibers and bacterial surface receptors (21, 22). After adhesion, phages inject their genetic material into the target bacterium, hijacking bacterial machinery for replication and maturation (23). Depending on the type of replication cycle, the fate of the phage and bacterial host is different. In the lytic cycle, the process ends violently for the bacterium, as the phage induces lysis—bursting the host open—resulting in the release of phage progeny and bacterial debris into the environment. Some phages have lysozymes in their tails and can cause lysis of susceptible bacteria without infection (24). Phages have many unique virtues, including that some can counter bacterial evolution with their own adaptation and thus can be harnessed via directed evolution to kill phage-resistant strains, a feature not possible with small molecule-based antibiotics (25, 26). They can be selected for unique antibacterial activities such as dispersion of biofilms (27) or the targeting to mucosal surfaces where bacteria reside (28). They also spare the microbiome, amplify at the site of infection (so-called “self-dosing”), and are generally regarded as safe by the FDA. According to current FDA requirements, phages intended for use in an Investigational New Drug application must not carry genes associated with antibiotic resistance or toxin production, nor exhibit lysogenic behavior (29, 30). Some groups have engineered phages to be therapeutic, while others have used phage lysins as novel antimicrobials (31, 32). As of now, the FDA has approved phages for therapeutic use under the expanded access program (30, 33). There have been numerous cases of phage therapy success documented and reviewed (19, 29, 34–36), as well as numerous clinical trials centered around phage therapy (30, 37).

Animal studies have investigated the use of bacteriophages for the treatment of bacteremia (bacteria in blood). Multiple groups have reported the effectiveness of intraperitoneal administration of phages in reducing bacterial load and mortality risk in rodent models of systemic infection from ESKAPE pathogens: *Enterobacter faecium* (38)*, S. aureus* (39, 40)*, Klebsiella pneumoniae* (41–44)*, Acinetobacter baumannii* (45–47)*, Pseudomonas aeruginosa* (41, 48–50), and *E. coli* (41, 51–53). Our group has demonstrated the efficacy of phage therapy in clearing *E. coli* bacteremia in murine models of infection (18, 25, 54). Despite all of these data, there are insufficient data on the acute and long-term immune responses following phage therapy (30). Previous studies have reported that clearance of bacterial infections requires both phages and the host immune system working in concert, using mathematical (55–57) and in vivo (49, 58) models. For instance, pathogen-associated molecular patterns (PAMPs) released after bacterial lysis can stimulate the innate immune system, complementing the bactericidal activity of phages, and thus, eradicating the infection (59). Phages also stimulate an adaptive immune response in the form of neutralizing anti-phage antibodies (60–65)—a concern for long-term phage treatment.

Our group collaborates with physicians by developing personalized phage cocktails against a patient’s untreatable AMR infection (19, 66). One interesting finding from these collaborations is that some patients with chronic *E. coli* infections that were treated with a unique cocktail of anti-*E. coli* phages seemed to not only have efficacious treatment responses to phage therapy but also remained infection-free for > 1 y or more. Since there was evidence that these patients had either an innate immune response upon phage therapy or developed neutralizing antibodies against phage, we hypothesized that they may have also developed an immune response against the original infecting bacterium after the administration of phage that was maintained after phage was gone. Here, we report an observation of successful treatment of lethal bacterial infection with phages in a murine model of bacteremia conferring protection against reinfection. Such a finding is consistent with a phage-stimulated long-term protective response, likely stemming from a stimulation of adaptive immunity against the original infecting bacterium. If true, the finding provides a shift in our understanding of phage therapy, implying that a phage may simultaneously be considered both a therapeutic and a method for conferring long-term immunity, a dual-action modality.

## Results

### Phage Therapy Drives Protection Against a Second Infection.

**Fig. 1.**
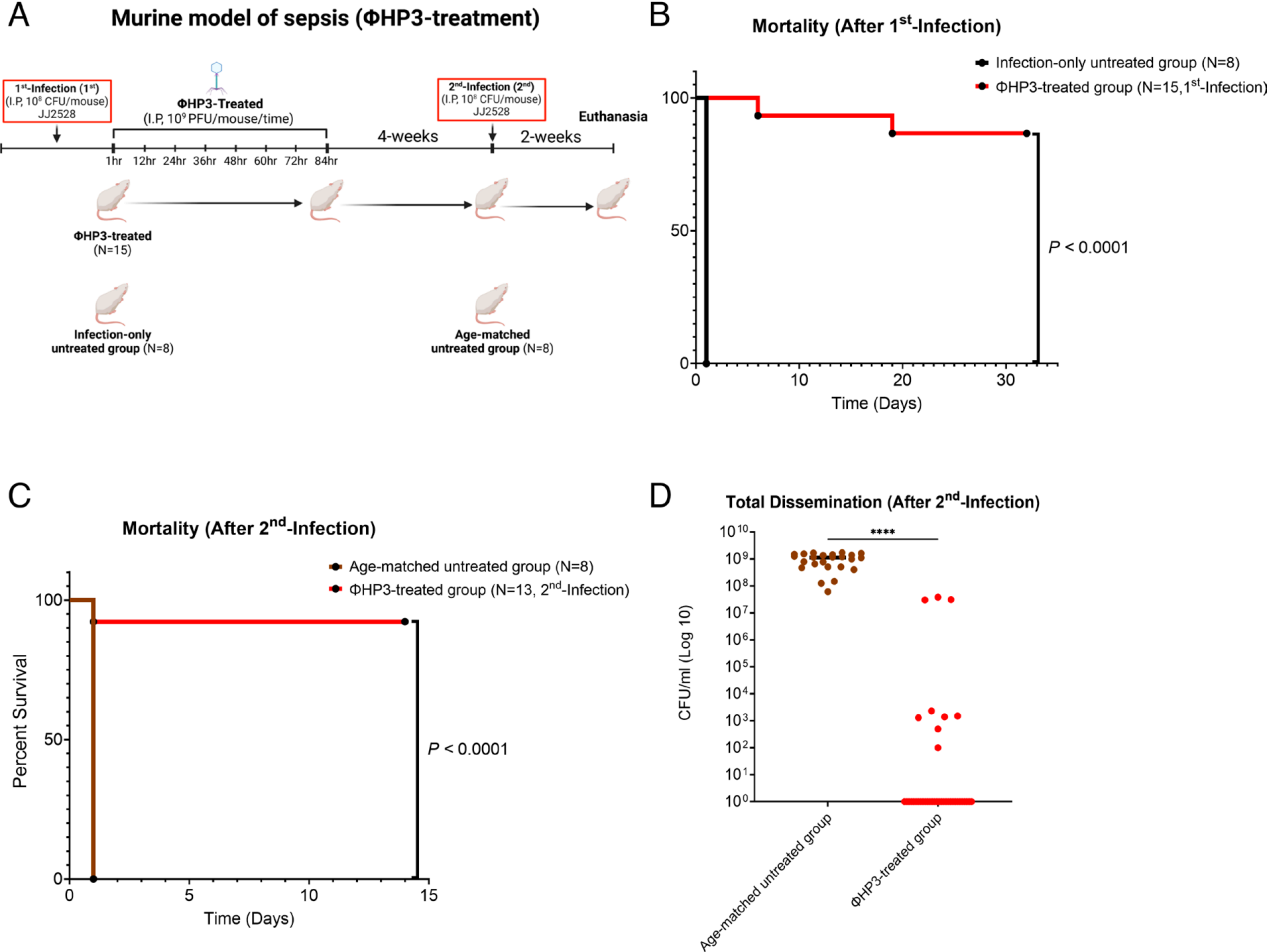
Evaluation of the therapeutic and protective efficacy of ΦHP3 against lethal ExPEC infection in a murine model of bacteremia. (*A*) Schematic of the murine bacteremia model with ΦHP3 treatment (created with BioRender.com). Female BALB/cJ mice were infected intraperitoneally with a 1 × 10^8^ CFU of JJ2528. ΦHP3-treated mice received 1 × 10^9^ PFU of ΦHP3 phage per mouse, administered eight times at 12 h intervals. 4 wk posttreatment, mice were rechallenged with 1 × 10^8^ CFU of JJ2528. 2 wk following rechallenge, mice were euthanized, and the liver, kidneys, and spleen were homogenized and plated to measure bacterial burden. (*B*) Survival rates following the 1st infection and (*C*) 2nd infection were analyzed using the log-rank (Mantel–Cox) test. (*D*) Median CFU/mL of JJ2528 after 2nd infection, combining counts from all organs.

To test the hypothesis that phage may induce a long-term protective response against a second infection, we adapted our previous model of bacteremia (18) for phage therapy using the therapeutic phage HP3 (ΦHP3). A T-4 like myovirus, ΦHP3 is a lytic phage that displays broad host range (67) and therapeutic efficacy in animal models (18, 54) and in compassionate used cases (19, 29, 66). In the initial study, mice were inoculated intraperitoneally with 1 × 10^8^ CFU of ExPEC JJ2528, a clinical virulent ST131 strain (1st infection). 1 h postinfection, the animals received phage therapy (1 × 10^9^ PFU of ΦHP3 every 12 h for 4 d given intraperitoneally) or did not (Fig. 1*A*). All mice in the infection-only control group died within 1 d postinfection (8/8), whereas over 85% of ΦHP3-treated mice survived (13/15, *P*-value, *P <* 0.0001), demonstrating the high therapeutic efficacy of ΦHP3 against ExPEC (Fig. 1*B*).

4 wk after phage treatment, surviving mice from the ΦHP3-treated group were rechallenged intraperitoneally with 1 × 10^8^ CFU of JJ2528 (2nd infection) and monitored for an additional 2 wk. Age-matched untreated mice served as controls for the phage-treated group to make sure the second challenge was in fact lethal. Upon second infection, all mice in the aged-matched group died within 1 d (8/8), mirroring the results from the initial control group. Rather remarkably, of the ΦHP3-treated mice that survived the initial (1st) challenge, 92% also survived the 2nd infection (12/13 survived, adjusted *P*-value, *P <* 0.0001), without additional phage treatment (Fig. 1*C*). Analyses of bacterial dissemination 14 d after the infection showed very little presence of infecting ExPEC (all organs, Fig. 1*D*, adjusted *P*-value, *P <* 0.0001), including organ-specific reductions (*SI Appendix*, Fig. S1), thereby corroborating the results of the survival studies.

To rule out the presence of residual phage in mice, we assayed for phages in different organs after initial treatment and before reinfection protection. A new cohort of animals (N = 10) was infected with ExPEC and treated with HP3 as before (*SI Appendix*, Fig. S2*A*). 2 d after the final treatment, two mice were euthanized, and HP3 was detected in the liver, kidney, and spleen, but not in blood (*SI Appendix*, Fig. S2*B*). All surviving mice (N = 8) lived up to day 32 and were rechallenged with JJ2528, except for two mice that were euthanized for phage detection. In contrast to the mice after final treatment, no phages were detected in any tissues on day 32 (*SI Appendix*, Fig. S2*C*). Similarly, no phages were detected in mice euthanized 2 wk post–second infection, on day 46 (*SI Appendix*, Fig. S2*D*).

To determine whether this observation was unique to phage HP3, we also tested a different phage in our bacteremia model with phage therapy. Here, another cohort of animals (N = 12) were infected with the same ExPEC strain and treated with the lytic phage Φ6954, which we recently discovered from wastewater. Phage 6954 is a podovirus, genomically, morphologically, and taxonomically distinct from ΦHP3, and allows us to determine whether a very different phage can induce a similar response. Although the therapeutic efficacy was not as protective as for ΦHP3 (*SI Appendix*, Fig. S2*E*), which argues for the importance of choosing the correct phage for therapy, all surviving mice were protected from a bacterial rechallenge without further treatment (*SI Appendix*, Fig. S2*F*). Collectively, these data suggest there is something about the initial phage treatment that provides significant protection against a second lethal infection.

### Phage Resistors Do Not Confer Protection against a Second Infection.

**Fig. 2.**
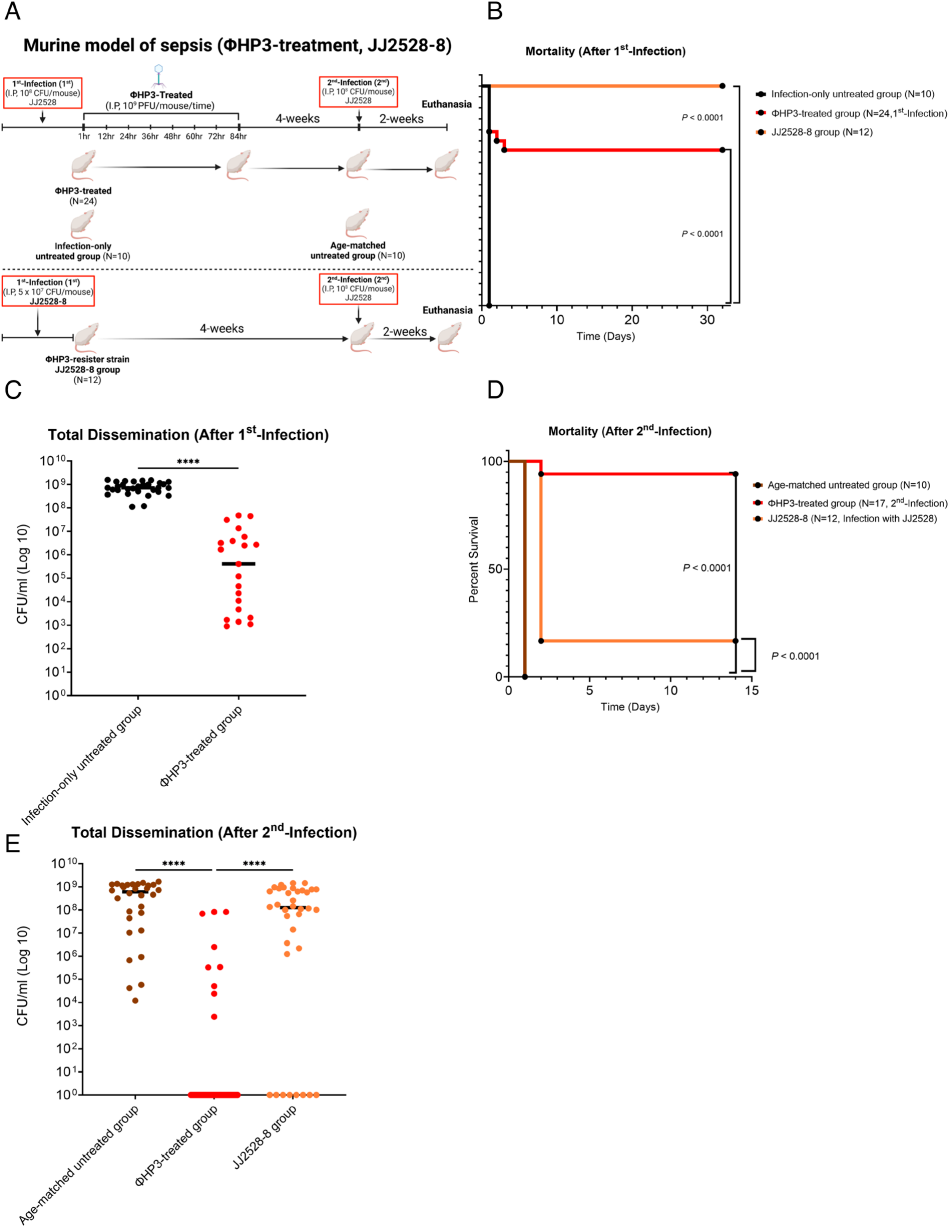
Evaluation of the protective efficacy of prior ΦHP3-resistant *E. coli* ExPEC JJ2528-8 infection against lethal ExPEC JJ2528 infection in a murine bacteremia model. (*A*) Schematic of the murine bacteremia model with the ΦHP3-resistant *E. coli* strain JJ2528-8 (created with BioRender.com). Female BALB/cJ mice were infected intraperitoneally with 1 × 10^8^ CFU of JJ2528. ΦHP3-treated mice received 1 × 10^9^ PFU of ΦHP3 phage per mouse, administered eight times at 12 h intervals. Mice in the JJ2528-8 group were infected intraperitoneally with 5 × 10^7^ CFU of the ΦHP3-resistant *E. coli* strain JJ2528-8 (LPS-truncated) instead. ΦHP3-treated mice received 1 × 10^9^ PFU of ΦHP3 phage per mouse, administered eight times at 12 h intervals. 4 wk posttreatment, all mice were challenged with 1 × 10^8^ CFU of JJ2528. 2 wk following challenge, mice were euthanized, and the liver, kidneys, and spleen were homogenized and plated to measure bacterial burden. (*B*) Survival rates following the 1st infection were analyzed using the log-rank (Mantel–Cox) test. (*C*) Median CFU/mL of JJ2528 after 1st, combining counts from all organs. (*D*) Survival rates following the 2nd infection were analyzed using the log-rank (Mantel-Cox) test. (*E*) Median CFU/mL of JJ2528 after 2nd infection, combining counts from all organs.

Our previous research demonstrated that phage-resistant bacterial strains emerge and persist after treatment with ΦHP3 in vivo (25). These resistors lose some portion of critical surface structures, including lipopolysaccharide and/or outer membrane protein A, thereby making them avirulent due to increased sensitivity to complement and susceptibility to phagocytosis (25). To explore whether the emergence and involvement of phage-resistant strains could confer protection against a secondary infection, we infected a cohort of animals with the ΦHP3-resistant strain ExPEC JJ2528-8 (LPS truncated, nonlethal) (25). This allowed us to test whether mice inoculated with a high dose [5 × 10^7^ CFU/mouse- based on previous published findings (25)] of the nonlethal, ΦHP3-resistant strain JJ2528-8 (derived from JJ2528) could also generate robust protection against a second lethal dose of parental [wild type (WT) ExPEC JJ2528. All other groups followed the previously established infection and treatment protocol (Fig. 2*A*). After the first infection with WT JJ2528, all mice in the infection-only (no phage treatment) control group died within 1 d postinfection (10/10), while approximately 71% of the ΦHP3-treated mice survived (17/24, *P <* 0.0001), mirroring our initial observation. In comparison, no fatalities occurred in mice infected with the ΦHP3-resistant strain JJ2528-8 (Fig. 2*B*), consistent with its attenuated nature. As above, the analysis of bacterial burdens in all organs (Fig. 2*C*) or each specific organ (*SI Appendix*, Fig. S3*A*) showed a strong dichotomy between the untreated and treated animals. When combining counts from all organs, the data revealed that deceased ΦHP3-treated mice exhibited significantly lower median ExPEC JJ2528 levels compared to infection-only controls after the first infection (*P <* 0.0001) (Fig. 2*C*).

4 wk posttreatment, all surviving mice from the ΦHP3-treated cohort and mice in the JJ2528-8 (attenuated) group were rechallenged with 1 × 10^8^ CFU of WT ExPEC JJ2528 (virulent). Survival rates were monitored and recorded for an additional 2 wk as previously described. Results indicated that all mice in the age-matched group died within 1 d postinfection (0/10 surviving), demonstrating once more the consistency of lethal challenge. Surprisingly, only 16.7% of mice in the JJ2528-8 initial infection group survived the 2 wk period (2/12 surviving), suggesting very limited protection provided by prior inoculation with the ΦHP3-resistant strain JJ2528-8. In contrast, over 90% of the ΦHP3-treated mice survived the 2nd infection (16/17 survived, *P <* 0.0001) (Fig. 2*D*). Additionally, combining bacterial burdens from all organs, the ΦHP3-treated mice exhibited a significant reduction in median ExPEC JJ2528 bacterial levels compared to the other two control groups (adjusted *P*-value, *P <* 0.0001; *P <* 0.0001), indicating strong protection in the ΦHP3-treated mice against a second infection. No significant difference was observed in bacterial burdens between the infection-only and resistor groups, with both maintaining consistently high levels (Fig. 2*E*). Organ-specific analysis also revealed significant decreases in bacterial loads of ΦHP3-treated mice compared to infection-only or resistor groups among all organs, respectively (*SI Appendix*, Fig. S3*B*). These results suggest that the emergence and involvement of the ΦHP3-resistant bacterial subpopulation, JJ2528-8, during ΦHP3 treatment does not confer protection against the second lethal infection and thus is unlikely to explain the long-term protection observed after phage therapy.

### Reinfection Protection Is Dependent on Phage Killing of ExPEC.

**Fig. 3.**
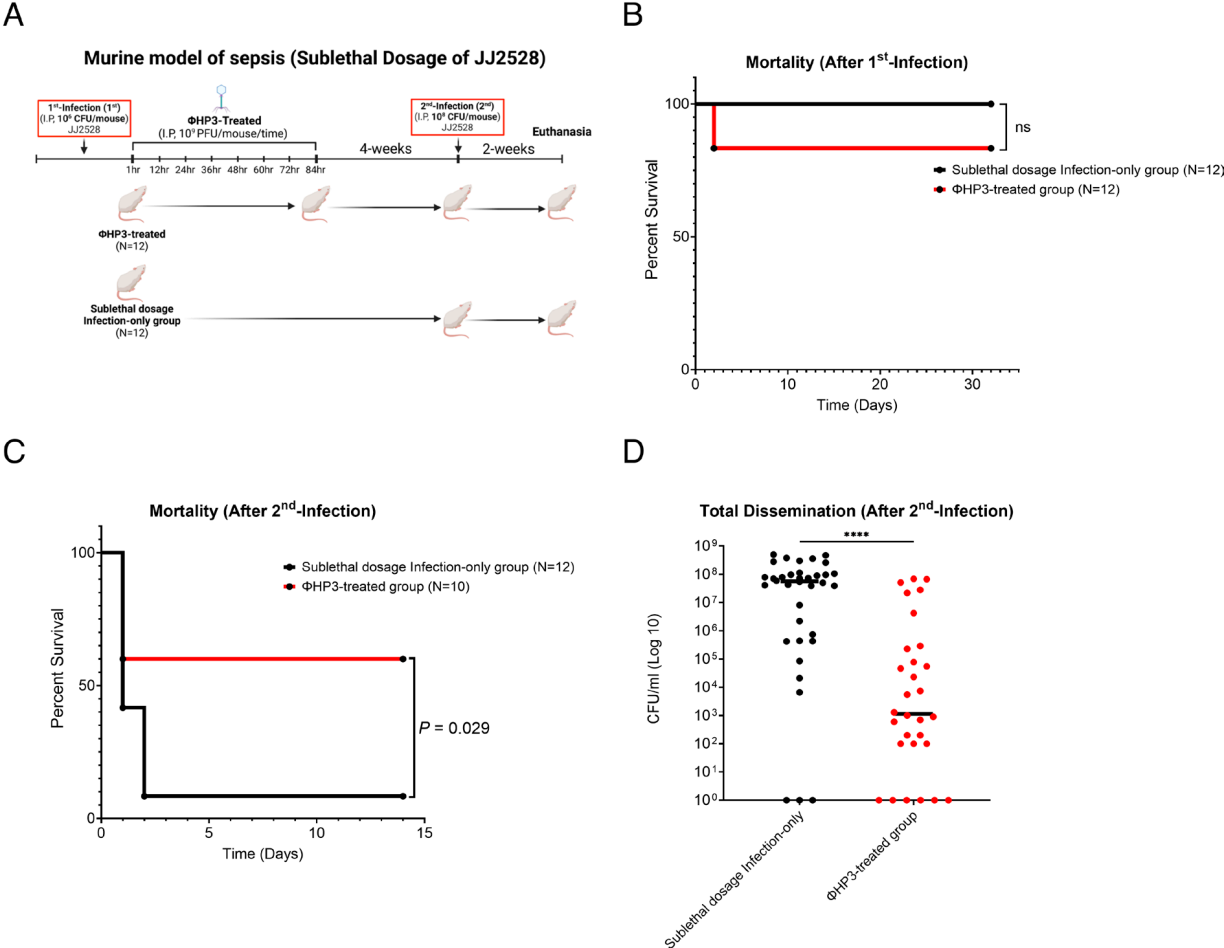
Evaluation of the protective efficacy of prior sublethal infection with ExPEC or ΦHP3-treated ExPEC sublethal infection against lethal ExPEC reinfection in a murine bacteremia model. (*A*) Schematic of the murine bacteremia model with the 1st infection using a sublethal dose of JJ2528, followed by a reinfection (2nd infection) with a lethal dose of JJ2528 (created with BioRender.com). Female BALB/cJ mice were infected intraperitoneally with 1 × 10^6^ CFU of JJ2528. ΦHP3-treated mice received 1 × 10^9^ PFU of ΦHP3 phage per mouse, administered eight times at 12 h intervals. 4 wk posttreatment, mice were rechallenged with 1 × 10^8^ CFU of JJ2528. 2 wk following rechallenge, mice were euthanized, and the liver, kidneys, and spleen were homogenized and plated to measure bacterial burden. (*B*) Survival rates following the 1st infection and (*C*) 2nd infection were analyzed using the log-rank (Mantel–Cox) test. (*D*) Median CFU/mL of JJ2528 after 2nd infection, combining counts from all organs.

Given that lingering phage ExPEC resistors cannot explain reinfection protection, we hypothesized that it may be induced by the actual lysing of the bacteria by phage. Because the animals quickly die in this model without treatment, we needed to create an experimental situation whereby the animals were stably infected but did not succumb to the infection so that they could be rechallenged after 30 d. We thus worked out conditions to find a sublethal dose of ExPEC JJ2528, settling upon 1 × 10^6^ CFU. As such, mice were infected as before with ExPEC JJ2528 (1st infection) and then either given phage ΦHP3 (1 × 10^9^ PFU, following the schedule described above) or untreated (Fig. 3*A*). All mice in the infection-only group survived, whereas two out of twelve in the ΦHP3-treated group succumbed (Fig. 3*B*). 4 wk later, all surviving mice, including those from the sublethal dosage infection-only control, were reinfected with 1 × 10^8^ CFU of JJ2528 (a now lethal dose), and their survival rates and bacterial levels monitored after 2 wk (or as animals became moribund). Rather surprisingly, following rechallenge of both groups, the ΦHP3-treated group showed a 60% survival rate (6/10 mice survived) while only a single animal in the phage untreated group (1/12, 8.3%) survived (adjusted *P*-value, *P* = 0.029—Fig. 3*C*). Consistent with previous observations, both the total (adjusted *P*-value, *P* < 0.0001, Fig. 3*D*) and organ specific ExPEC levels (*SI Appendix*, Fig. S4) were lower in the cohort initially treated with phage despite no further phage treatment upon challenge. Taken together, these data indicate that previous nonlethal infection is not sufficient to explain the long-term protection against a second lethal infection conferred by phage treatment.

### Reinfection Protection Is Dependent on Phage Lysis.

**Fig. 4.**
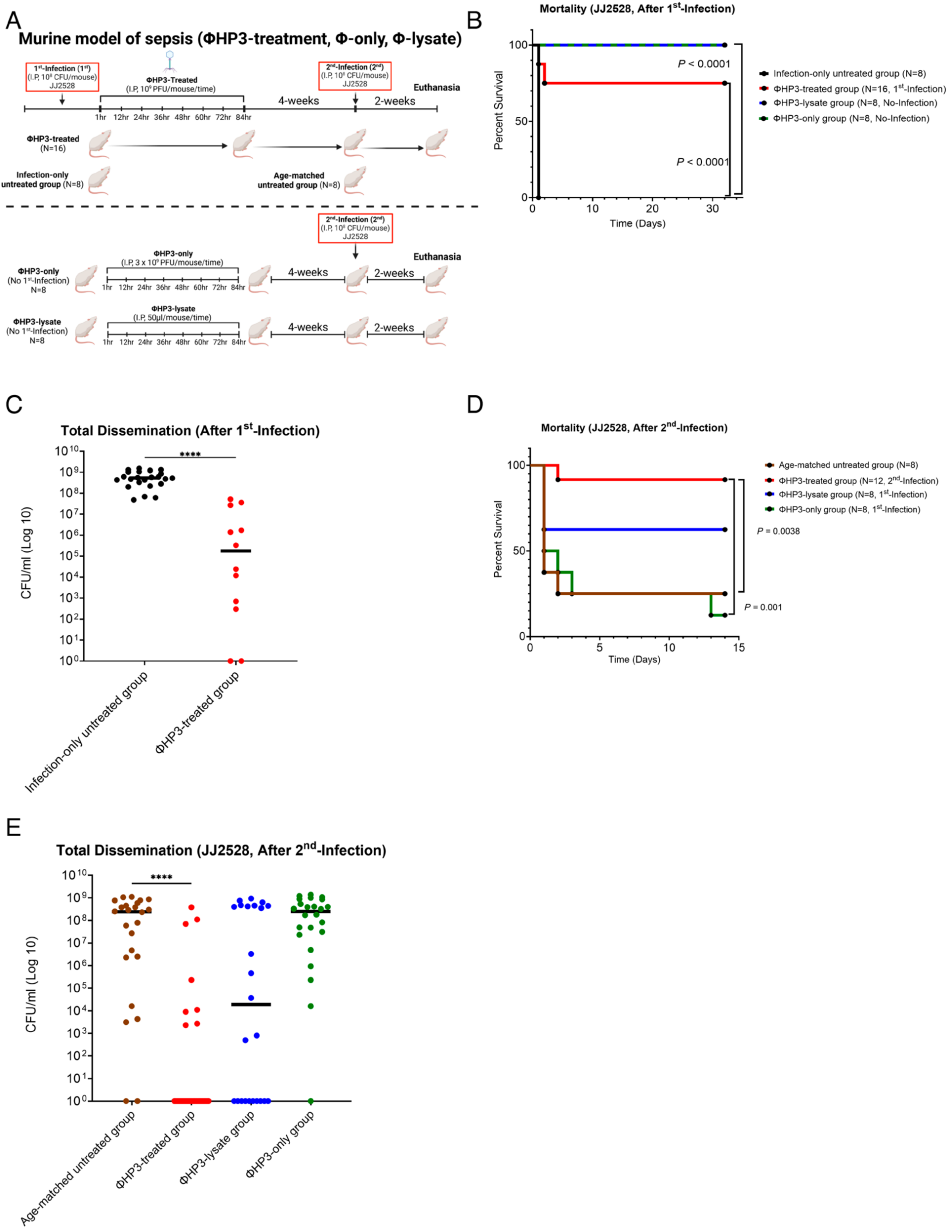
Evaluation of the protective efficacy of ΦHP3-only and ΦHP3-lysate against lethal ExPEC infection in the murine model of bacteremia. (*A*) Schematic of the murine bacteremia model of ΦHP3-lysate and ΦHP3-only (created with BioRender.com). Female BALB/cJ mice were infected intraperitoneally with a 1 × 10^8^ CFU of JJ2528, except for the ΦHP3-only and ΦHP3-lysate groups. ΦHP3-treated and ΦHP3-only mice received either 1 × 10^9^ PFU or 3 × 10^9^ PFU of ΦHP3 phage per mouse, respectively, administered eight times at 12 h intervals. ΦHP3-lysate mice received 50 μL of ΦHP3-lysate (*Materials and Methods*) per mouse administered eight times at 12 h intervals. 4 wk posttreatment, all mice were challenged with 1 × 10^8^ CFU of JJ2528. 2 wk following challenge, mice were euthanized, and the liver, kidneys, and spleen were homogenized and plated to measure bacterial burden. (*B*) Survival rates following the 1st infection were analyzed using the log-rank (Mantel–Cox) test. (*C*) Median CFU/mL of JJ2528 after 1st infection, combining counts from all organs. (*D*) Survival rates following the 2nd infection were analyzed using the log-rank (Mantel-Cox) test. (*E*) Median CFU/mL of JJ2528 after 2nd infection, combining counts from all organs.

The above studies suggest that some combination of phage + bacteria is important to confer reinfection protection on the murine host. To discern whether the lytic action of the phage (compared to the phage itself) was driving this response, we introduced two additional cohorts into the study: One receiving only phage ΦHP3 (ΦHP3-only) and another receiving the phage-generated ExPEC JJ2528 lysate (ΦHP3-lysate). The ΦHP3 lysate is meant to approximate the lysis expected during phage therapy in vivo (Fig. 4*A*). Although in vitro and in vivo lysis by phage may present molecular differences that we are not aware of, we did try to keep the levels of phage and bacteria in the reaction stoichiometrically as close to what is delivered during treatment and/or infection so that a direct comparison can be made (*Materials and Methods*). Mice in the infection-only untreated and ΦHP3-treated groups were first infected with a lethal dose of ExPEC as before. The ΦHP3-treated and ΦHP3-only groups were administered ΦHP3 (1 × 10^9^ PFU/mouse), a total of eight times every 12 h. Finally, the ΦHP3-lysate group received 50 uL of the phage-generated ExPEC lysate, following the same dosing regimen as the phage-administered animals. Following the initial infection, 75% of the ΦHP3-treated mice survived (12/16 mice survived) (adjusted *P*-value, *P* < 0.0001, Fig. 4*B*). Consistent with previous experiments, all animals that were not treated with phage died. As expected, no ΦHP3-only or ΦHP3-lysate-treated mice died. Phage treatment also reduced ExPEC levels as before (adjusted *P-*value, *P <* 0.0001, Fig. 4*C* and *SI Appendix*, Fig. S5*A*).

4 wk later, as before, all cohorts were challenged with a lethal dose of ExPEC. After the second infection, the previously phage-treated animals showed robust protection against ExPEC rechallenge (adjusted *P-*value, *P* = 0.0038, Fig. 4D, 92%, 11/12 mice surviving). In contrast, the ΦHP3-only control group and the age-matched group were not protected, with survival rates of around 13% (1/8 mice survived) and 25% (2/8 mice survived), respectively. This suggests that phage ΦHP3 alone does not account for the protection against a second lethal infection. Notably, the ΦHP3-lysate group demonstrated more protection (60%, 5/8 mice survived). The ΦHP3-treated mice demonstrated a significant reduction in median ExPEC JJ2528 levels compared to infection-only controls (adjusted *P-*value, *P <* 0.0001, Fig. 4*E* and *SI Appendix*, Fig. S5*B*), and while there was a trend toward reduction for the ΦHP3-lysate group, it was not statistically significant compared to the infection-only group. Others have reported that bacterial lysates can act as potent immunizing agents (68–72). In general, the practice is discouraged because the lysates are uncharacterized and may contain harmful toxins or pyrogens. To compare the level of protection provided by more historically traditional immunization methods, mice (N = 10) were inoculated with a mechanical lysate of JJ2528 (*Materials and Methods*) following the same treatment schedule as the ΦHP3-only and ΦHP3-lysate groups (*SI Appendix*, Fig. S6*A*). This group was not challenged with ExPEC at the start of the experiment, as we expected all mice to succumb to infection due to the lack of therapeutic value of the lysate. 4 wk postinoculation, the mechanical lysate-treated mice were challenged with JJ2528 for the first time, and 90% survival was observed (*SI Appendix*, Fig. S6*B*), suggesting that inoculation with cells lysed mechanically also generates a lasting adaptive response that protects against reinfection. Since a phage-generated lysate showed an intermediate level of protection against both lethal rechallenge and ExPEC dissemination, these data suggest that some of the long-term protection is driven in part by the lytic activity of the phage against its target and not the phage itself.

## Discussion

In this report, we describe a phenomenon whereby the previous treatment of a bacterial infection with phage therapy induces a long-term protective effect against subsequent infection with a lethal dose of the same bacterial strain. We report a demonstration of this effect. There is strong scientific evidence that this observation is real, at least with this phage (HP3) in this model system, and at least one other very different phage (6954). To validate the point about the evidence being strong, it is important to consider all the data together. For example, when the data from all cohorts are aggregated, phage treatment results in ~76% of the animals surviving a lethal infection, and a 100-fold reduction in total and organ-specific bacterial burden (N = 55 animals, *SI Appendix*, Fig. S7 *A–C*). In contrast, without phage, not a single animal lived past 24 h (N = 26), demonstrating the strong protective effect of phage therapy. Rather remarkably, the long-term protective effect was even stronger. Of the 42 animals that survived the first infection after being given phage therapy, 93% (39/42) survived a lethal rechallenge of ExPEC in the absence of additional phage therapy. Only 2 of 26 age-matched controls survived infection. We are including these data in the supplement so the audience can appreciate the strong reproducibility and rigor of these studies (*SI Appendix*, Fig. S7 *D–F*). No evidence of phage could be detected at this point (*SI Appendix*, Fig. S2.), leading us to conclude that reinfection protection is likely due to some type of immunization of the animals because of phage therapy. This is further supported by the finding that reinfection protection is not observed with just phage, lingering attenuated phage resistors, or a lingering sublethal bacterial infection but is partially observed with a phage generated lysate, suggesting the mechanism of protection is stimulated by the lytic activity of phage itself. We term this activity phage-induced immunization or ΦII.

The immune response to pathogens is often divided into either innate or adaptive immunity. There is strong evidence in the literature of innate immune response to phage. Over the past decade, multiple reviews have examined the interaction between phages and the immune system (73–76). Phages possess PAMPs—DNA, RNA, and various proteins that can activate pattern recognition receptors including Toll-like receptors (76). In fact, phage capsid proteins are reported to exhibit adjuvating properties by activating the innate immune system (77, 78). Reported cytokine responses to bacteriophages range from pro- to anti-inflammatory (76). Although contamination with bacterial debris in the phage preparations might account for strong responses in some studies (79, 80), the reported variability in responses might be phage-dependent (76). Administered systemically, phages can elicit the production of specific anti-phage antibodies in the host (60–65) which may reduce phage activity and hinder the outcome of treatment (64, 81). The therapeutic efficacy of phage therapy comes not only from the lytic killing of bacteria but also from the induction of an antibacterial immune response by phages or bacterial components (82). For example, neutrophils were required for successful treatment with phages against a *P. aeruginosa* lung infection in mice (58). A different study demonstrated that phages also enhance phagocytosis (83). Finally, others have suggested that PAMPS released during phage-mediated bacterial lysis can further stimulate the immune system, helping with bacterial clearance (59). Hence, some phages do not act alone in clearing bacterial infections but work in concert with the host innate immune system in the moments immediately after therapy.

In this report, we provide evidence of a long-term protective response that looks remarkably like adaptive immunity. We hypothesize that the lysis of bacterial cells by phage (ΦHP3) releases previously cryptic antigens that, because they are now released and abundant, are recognized and taken up by phagocytic cells. The antigens are unknown but could be linked to release of either novel antigens from the hydrolysis of surface peptidoglycan, membrane, or lipopolysaccharide by lysins or intracellular antigens leaked via the bursting of bacterial cells or both. We cannot rule out that novel structural epitopes of known proteins are also exposed or that virulence factors are released when previously they were controlled or hidden. There may also be multiple protective antigens that provide a combinatorial benefit. We speculate these antigens are processed for B-cell development and result in a plethora of either opsonophagocytic or neutralizing antibodies or both.

Because the protection is strong, it is likely these antibodies target either surface components or important virulence factors. We cannot rule out and think it likely that the phage itself acts as a kind of adjuvant. As pointed out above, some phages are known to induce innate adaptive responses. This stimulation of the immune system may further drive APC recruitment and antigen uptake and processing, thereby driving strong protective adaptive responses. Because our data indicate that this response is not only dependent on the phage structure and its mechanism of lytic killing, we predict a gradient in a phage’s ability to induce immunity, likely dependent on PAMPs it possesses and its specific lytic enzymology. Thus, we urge those considering phage therapy to screen for phages that possess this activity. Because such work may require extensive effort, and due to the potential importance of this finding for phage therapy, we felt it important to report.

Our study does have some limitations. First, the exact mechanism of protection is not known.

We are currently examining several hypotheses. On this front, it is reasonable to assume the data are consistent with phage therapy inducing an adaptive or humoral immune response that extends beyond its initial lytic effects. This adaptive response would protect upon subsequent exposures to the same bacterial pathogen, much like a vaccination. Although we will be describing our efforts to understand this mechanism in future studies, we provide a reasonable explanation here as to what may be happening. ExPEC, like other human-adapted pathogens, is a master at keeping critical antigens either masked or nonimmunogenic (16, 84). Phage may release these antigens for processing. Second, it will be intriguing to know how long protection extends, that is, the duration of the response. Third, we do not know whether other phages can perform this activity or whether the protection extends to other ExPEC strains that differ in genotype and phenotype. Our ongoing work suggests the latter to be true, but the breadth of protection beyond the original strain is still unclear and might be influenced by the infecting strain. We are exploring these ideas currently, as well as the molecular mechanism that underlies this effect.

Our findings have several implications. First, the effect may be a type of personalized vaccination. Many of the ESKAPE pathogens are masters of genetic change, mix and matching virulence factors. This makes a silver bullet approach using a single virulence factor that covers all strains as a vaccine difficult to achieve. However, if phages can be selected with broad host ranges (or a cocktail) that covers most circulating strains, if they kill the target and have immunogenic properties, regardless of the genetic makeup of the infecting bacterial strain, they may induce vaccination. Second, since many chronic infections are plagued by these bacteria hiding in microbiome reservoirs, essentially causing reinfection of the host, if the body is trained via phage lysis of the target to recognize them, it may prevent reinfection from these reservoirs. This could be particularly important for preventing recurrent UTI or lung infections. Third, it may open the door to biotechnology applications of phages, especially if the very antigens deemed protective are themselves engineered to be encoded and expressed by the infecting phage, a strategy that should amplify the strength and duration of the adaptive response. It is also not lost on the authors that phage-induced immunity at the mucosal surface (where bacteria often live) with phages that specifically direct to these areas might be a mechanism to induce local mucosal immunity, especially in the bladder and GI tract.

## Materials and Methods

### Ethics Statement.

All methods performed on mice were conducted in accordance with relevant guidelines and regulations from “The Guide for the Care and Use of Laboratory Animals” (NIH). The Animal Use Protocol number AN-6372 was approved by Baylor College of Medicine’s Institutional Animal Care and Use Committee.

### Bacterial Strains and Culture Conditions.

The *E. coli* strains used in this study were cultured overnight from a single colony in Lysogeny broth plate (LB; 10 g/l tryptone, 0.5 g/l sodium chloride (NaCl), and 5 g/l yeast extract) at 37 °C after resuscitation from a frozen stock (−80 °C, 10% glycerol). The ExPEC ST131 strains used in the study, JJ2528, were kindly provided by James R. Johnson (85). The ΦHP3-resister strain JJ2528-8 (LPS-truncated), utilized in one of our murine sepsis models, originated from our earlier research (25). The DH5α Competent *E. coli* strain was used for phage purification. The number of colony-forming units (CFU) administered was determined by correlating the optical density (OD) at 600 nm to the number of colonies observed after plating.

### Experimental Animals.

6-wk-old female BALB/cJ mice used in this study were obtained from Jackson Laboratories (Bar Harbor, ME). They were provided with sterile food and water ad libitum and housed in filtered cages with 3 to 4 mice per cage. All experimental procedures performed on mice were approved in accordance with relevant guidelines and regulations from “The Guide for the Care and Use of Laboratory Animals” (NIH) and were approved by Baylor College of Medicine’s Institutional Animal Care and Use Committee under protocol number AN-6372.

### Phage Purification and ΦHP3-Lysate Preparation.

The phage HP3, previously isolated (18) and characterized (67), was purified by cesium chloride gradient, as described before (18). For the preparation of the phage-generated bacterial lysate (ΦHP3-lysate), 50 mL of LB media was inoculated with a 1:100 dilution of an overnight culture of JJ2528 and incubated with shaking at 37 °C until the optical density at 600 nm reached 0.1 to 0.2. The culture was then infected with ΦHP3 at a multiplicity of infection of 1 and continued to incubate with shaking at 37 °C until the culture became clear. The resulting bacterial lysate was stored at 4 °C.

### Phage Treatment and Administration of ΦHP3-Only or ΦHP3-Lysate.

In the ΦHP3-treated group, mice received an intraperitoneal injection (I.P.) of 1 × 10^9^ PFU of ΦHP3 per mouse 1 h after the initial infection (1st infection). This regimen was repeated every 12 h for a total of eight doses from Day 1 to Day 4. For functional control groups, such as the ΦHP3-only and ΦHP3-lysate (a lysate of ExPEC strain JJ2528 lysed through lytic phage ΦHP3) groups, mice were treated with either 1 × 10^9^ PFU/mouse of ΦHP3 or 50 μL of ΦHP3-lysate per mouse (intraperitoneal injection), respectively, without first infection (1st infection). These treatments were also administered eight times at 12 h intervals from Day 1 to Day 4, mirroring the schedules of the ΦHP3-treated group. For all other types of phage treatments, the preparation and treatment procedures were identical to those described above.

### Murine Sepsis Model with Phage Therapy.

The *E. coli* strain JJ2528 was cultured overnight under specified conditions 1 d prior to injection. On the day of injection (Day 1), strains were subcultured in LB broth at a 1:100 dilution to an optical density at 600 nm (OD600) of approximately 0.6 (log phase, ~1 × 10^8^ CFU/mL), harvested by centrifugation (3,500×*g* for 20 min at 4 °C, Centrifuge 5702 R, Eppendorf North America, Framingham, MA), and resuspended in an equivalent volume of 1 × PBS. Mice in the infection-only and ΦHP3-treated groups were intraperitoneally injected with 50 μL of the bacterial suspension (1 × 10^8^ CFU), and the inoculum was quantified by plating dilutions onto LB agar. 1 h later, mice in the ΦHP3-treated group were administered 1 × 10^9^ PFU of ΦHP3 phage per mouse, repeated eight times at 12 h intervals. During the first week post–initial infection, mice were monitored twice daily, then once daily until the second infection. Survival rates were continuously monitored and recorded. 4 wk posttreatment, all surviving ΦHP3-treated mice and aged-matched mice were infected with 1 × 10^8^ CFU of JJ2528 and monitored twice daily for an additional 2 wk, with survival data collected continuously. After 1st and 2nd bacterial challenges, mice were euthanized and necropsied if found moribund [using the previous scoring system (18)] or at the end of the experiment. To determine bacterial levels (CFU/mL), the liver, kidneys, and spleen were collected and placed into 1 mL of 1× PBS, homogenized via bead beating with a BeadBlaster Refrigerated Homogenizer (Benchmark Scientific Inc, Sayreville, NJ), plated on LB agar plates, and incubated at 37 °C.

### Murine Sepsis Model with Phage-Resistant *E. coli*.

Following previous protocols, *E. coli* strain JJ2528 was cultured and prepared both 1 d prior to and on the day of injection. On Day 1, mice in the ΦHP3-treated group and infection-only group were intraperitoneally injected with 50 μL of the WT JJ2528 strain suspension (1 × 10^8^ CFU). 1 h later, mice in the ΦHP3-treated group received an intraperitoneal injection of 1 × 10^9^ PFU/mouse of ΦHP3. A functional control group was infected with the *E. coli* ΦHP3-resister JJ2528-8 strain (LPS-truncated) instead of the WT JJ2528. The preparation of the JJ2528-8 strain followed the same protocol as JJ2528. These mice were intraperitoneally injected with 50 μL of the JJ2528-8 suspension (5 × 10^7^ CFU). Following the initial (1st) infection, the survival rate of all mice was monitored and recorded as previously described. Between the two infections, moribund animals were euthanized and subjected to necropsy to evaluate bacterial loads in the kidneys, spleen, and liver. 4 wk posttreatment, all surviving mice and aged-matched mice were administered 1 × 10^8^ CFU/mouse of WT JJ2528 and were monitored twice daily to assess survival rates and bacterial burdens in various organs. 2 wk after the 2nd infection, all mice were euthanized, and bacterial levels were determined as previously described.

### Murine Sepsis Model with Sublethal Dosage Infection and Phage Therapy.

Following the established protocol, mice in the ΦHP3-treated group and the infection-only group were initially infected (1^st^ infection) with a sublethal dose of JJ2528 (10^6^ CFU/mouse). Subsequently, the ΦHP3-treated group received 10^9^ PFU/mouse 1 h postinfection, repeated every 12 h for a total of eight doses. 4 wk after treatment, all surviving mice, including those from the sublethal dosage of the JJ2528-infected group, were reinfected with a lethal dose of JJ2528 (10^8^ CFU/mouse). Monitoring occurred twice daily for an additional 2 wk, during which survival data were continuously collected. Moribund or deceased mice were euthanized and necropsied to assess bacterial levels in their organs using previously described methods. 2 wk post 2nd infection, all remaining mice were euthanized and necropsied to determine bacterial burdens in their organs.

### Murine Sepsis Model: ΦHP3 Treatment, ΦHP3 Lysate, ΦHP3 Only.

Mice in the ΦHP3-treated and infection-only control groups were infected with JJ2528 (I.P, 1 × 10^8^ CFU/mouse). Subsequently, mice in these groups received treatments with ΦHP3 (1 × 10^9^ PFU/mouse), as outlined earlier. The ΦHP3-Lysate and ΦHP3-only groups were administered treatments without prior (1st) infection as previously described. Survival of all mice was continuously monitored and recorded; moribund or deceased mice were necropsied, homogenized, and CFU determination. 4 wk posttreatment, all surviving mice, along with the 2nd infection untreated control group, were infected with JJ2528 (I.P, 1 × 10^8^ CFU/mouse). All mice were monitored twice daily to evaluate survival rates and assess bacterial burdens in various organs of moribund or deceased mice, following the procedures outlined in the previous model. 2 wk after the 2nd infection, all mice were euthanized, and bacterial levels were determined as previously described.

### Phage Detection.

To determine the level of phages during the experiment, we randomly selected and euthanized two mice in the HP3-treated group 2 d after the last treatment. We collected blood, liver, kidneys, and spleen and assayed these tissues for the presence of phages. With the exception of blood, we homogenized the organs as done elsewhere in our manuscript and centrifuged the homogenate at 13.3 rpm for 30 s. Following this, we serially diluted blood and organ homogenate supernatant in phage buffer (6.7 mM Tris-HCl, 3.2 mM Tris-base, 100 mM NaCl, and 10 mM MgSO^4^ 7H_2_O, pH 8.0, filter sterilized)—same buffer used during phage purification. Finally, we spotted 5 μL of each dilution and neat onto lawns of DH5α in a double agar overlay assay and incubated overnight at 37 °C

### Mechanical Lysate Preparation and Immunization.

*E. coli* strain JJ2528 was prepared as described above, and 1 × 10^8^ CFUs were lysed via bead beating (homogenized using 0.1 – mm-diameter glass beads for 5 min at 4 °C). Mice received an intraperitoneal injection (I.P.) of 50 μL of lysate per mouse, administered eight times at 12 h intervals from Day 1 to Day 4, without infection at the start of the experiment.

### Statistical Analyses.

We performed our statistical analyses using GraphPad Prism (GraphPad Software, San Diego, CA). To compare survival rates following first and second bacterial infections, we generated Kaplan–Meier survival curves and determined statistical significance by the log-rank (Mantel–Cox) test. We analyzed bacterial levels between two or more experimental groups by the Mann–Whitney U test or Kruskal–Wallis ANOVA with Dunn’s multiple comparisons, respectively, and used scatter dot plots for visualization. For bacterial levels, the detection limit was Log (2) = 100 CFU/mL. If no colonies were detected, the CFU count was adjusted to Log (1) = 0. Significance is marked by *P < 0.05, **P < 0.01, ***P < 0.001, and ****P < 0.0001.

## Supplementary Material

Appendix 01 (PDF)

## Data Availability

All study data are included in the article and/or *SI Appendix*.
