# Supplementary material for: Phage-induced protection against lethal bacterial reinfection
Source: Proc Natl Acad Sci U S A. 2025 May 30;122(22):e2423286122. doi: 10.1073/pnas.2423286122 (PMC12146767; doi:10.1073/pnas.2423286122)
Supplement: Supplementary file 1 — Appendix 01 (PDF) [file pnas.2423286122.sapp.pdf]

PNAS

Supporting Information for
Phage-induced Protection Against Lethal Bacterial Re-Infection

Yikun Xing[a,b,1], Haroldo J. Hernandez Santos[a,b,1], Ling Qiu[b], Samantha R. Ritter[a,b], Jacob J. Zulk[a,b], Kathryn A. Patras[b,c], Austen L. Terwilliger[a,b] and Anthony W. Maresso[a,b,*].

[a]TAILOR Labs, Vaccine Development Group, Baylor College of Medicine, Houston, TX 77030

[b]Department of Molecular Virology and Microbiology, Baylor College of Medicine, Houston, TX 77030

[c]Alkek Center for Metagenomics and Microbiome Research, Baylor College of Medicine, Houston, TX 77030

[1]Y.X. and H.H.S. contributed equally to this work.

*Anthony W. Maresso
Email: maresso@bcm.edu

This PDF file includes:

Figures S1 to S7

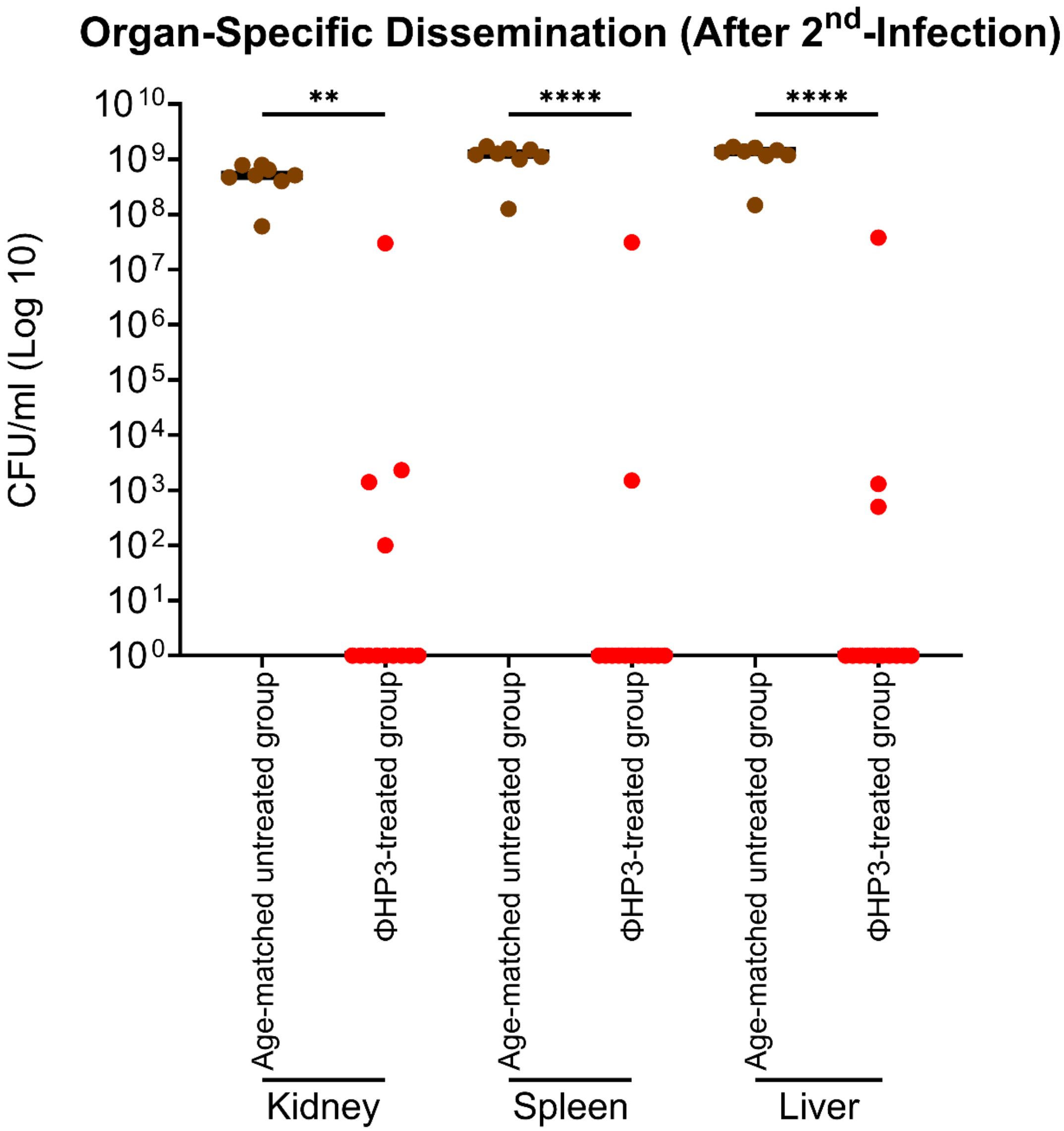

Fig. S1. Organ-specific analysis of bacterial dissemination in mice following second infections after ΦHP3 treatment.

Female BALB/cJ mice were infected intraperitoneally with a 1×10^8 CFU of JJ2528. ΦHP3-treated mice received 1×10^9 PFU of ΦHP3 phage per mouse, administered eight times at 12-hour intervals. Four weeks post-treatment, mice were re-challenged with 1×10^8 CFU of JJ2528. Two weeks following re-challenge, mice were euthanized, and liver, kidneys, and spleen were homogenized and plated to measure bacterial burden. Median CFU/mL of JJ2528 by organ following 2nd infection.

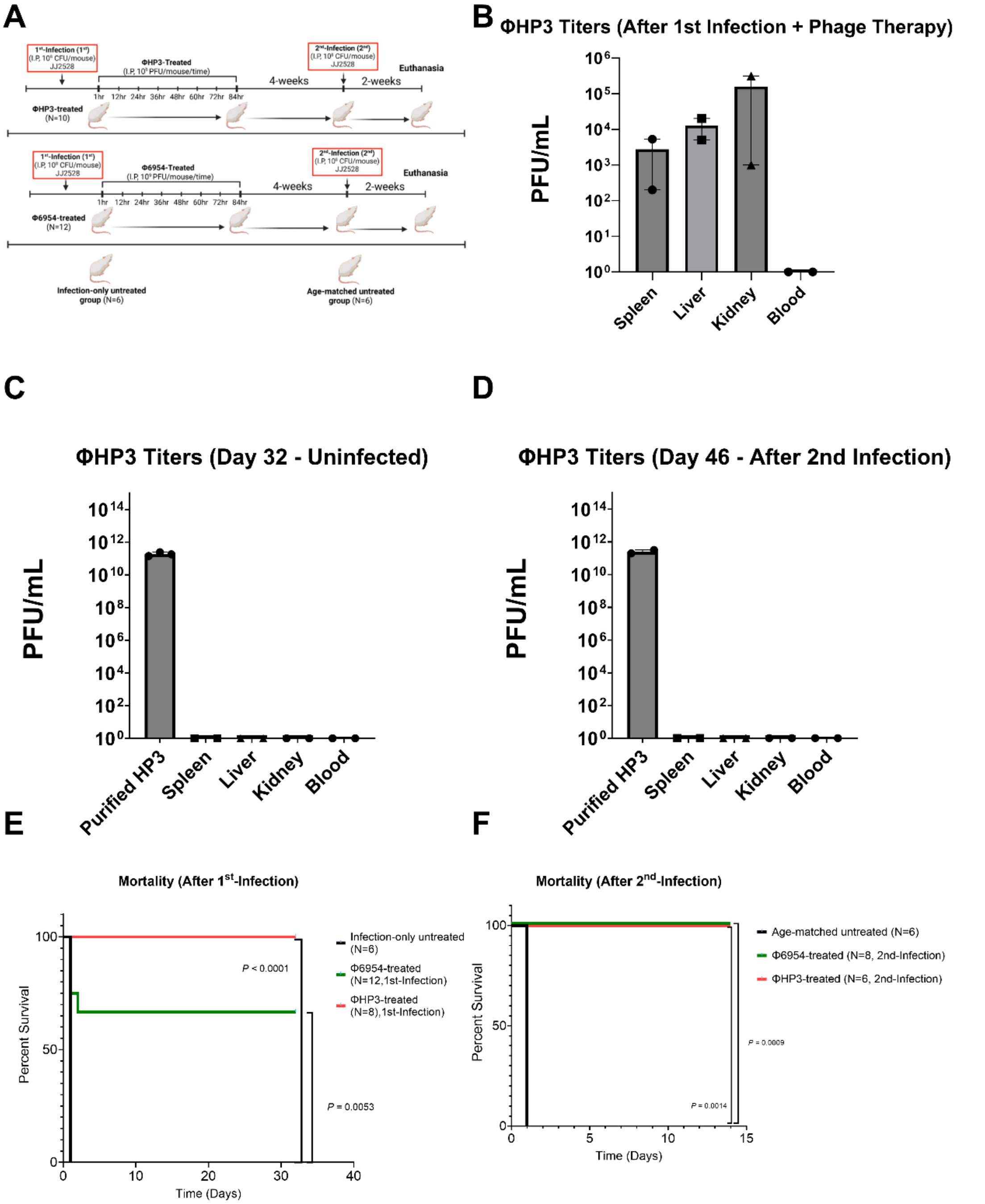

Fig. S2. Assessment of therapeutic and protective efficacy of ΦHP3 and Φ6954 against lethal ExPEC infection in a murine model of bacteremia.

(A) Schematic of the murine bacteremia model with phage treatment (created with BioRender.com). Female BALB/cJ mice were infected intraperitoneally with a 1 × 10^8 CFU of JJ2528. Phage-treated mice received 1 × 10^9 PFU of either ΦHP3 or Φ6954 per mouse, administered eight times at 12-hour intervals. Four weeks post-treatment, mice were re-challenged with 1 × 10^8 CFU of JJ2528. Two days after phage treatment and on day 32 before rechallenge, two animals from the ΦHP3-treated cohort at each time point were sacrificed,

organs homogenized, and phage HP3 levels determined using a double-agar overlay assay. The same procedure was applied to all surviving animals on day 46. The same infection-only untreated and age-matched untreated mice were used in Figure S6. Purified HP3 was used as a positive control on days 32 and 46. (**B)** ΦHP3 titers (PFU/mL) against *E. coli* DH5α two days after last phage treatment, (**C)** on day 32, and **(D)** on day 46. **(E)** Survival rates following the 1st infection and **(F)** 2nd infection were analyzed using the Log-rank (Mantel-Cox) test

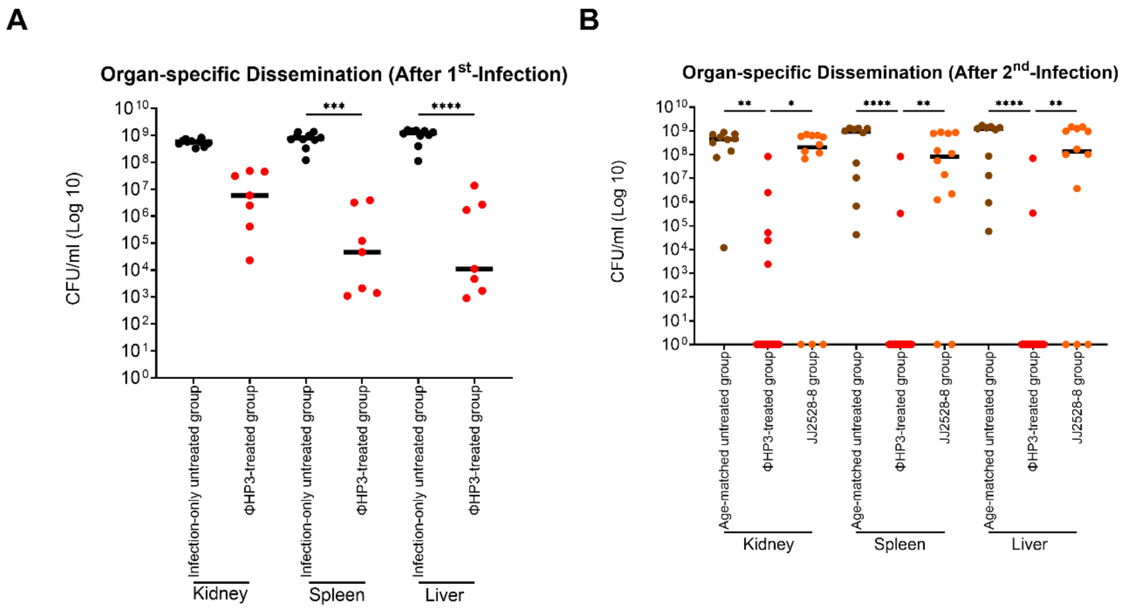

Fig. S3. Organ-specific analysis of bacterial dissemination in mice following infection with wild-type or mutant phage-resistant ExPEC.

Female BALB/cJ mice were infected intraperitoneally with 1×10^8 CFU of JJ2528. ΦHP3-treated mice received 1×10^9 PFU of ΦHP3 phage per mouse, administered eight times at 12-hour intervals. Mice in the JJ2528-8 group were infected intraperitoneally with 5×10^7 CFU of the ΦHP3-resistant *E. coli* strain JJ2528-8 (LPS-truncated) instead. ΦHP3-treated mice received 1×10^9 PFU of ΦHP3 phage per mouse, administered eight times at 12-hour intervals. Four weeks post-treatment, all mice were challenged with 1×10^8 CFU of JJ2528. Two weeks following challenge, mice were euthanized, liver, kidneys, and spleen were homogenized and plated to measure bacterial burden **(A)** Median CFU/mL of JJ2528 by organ following 1st and **(B)** 2nd infection.

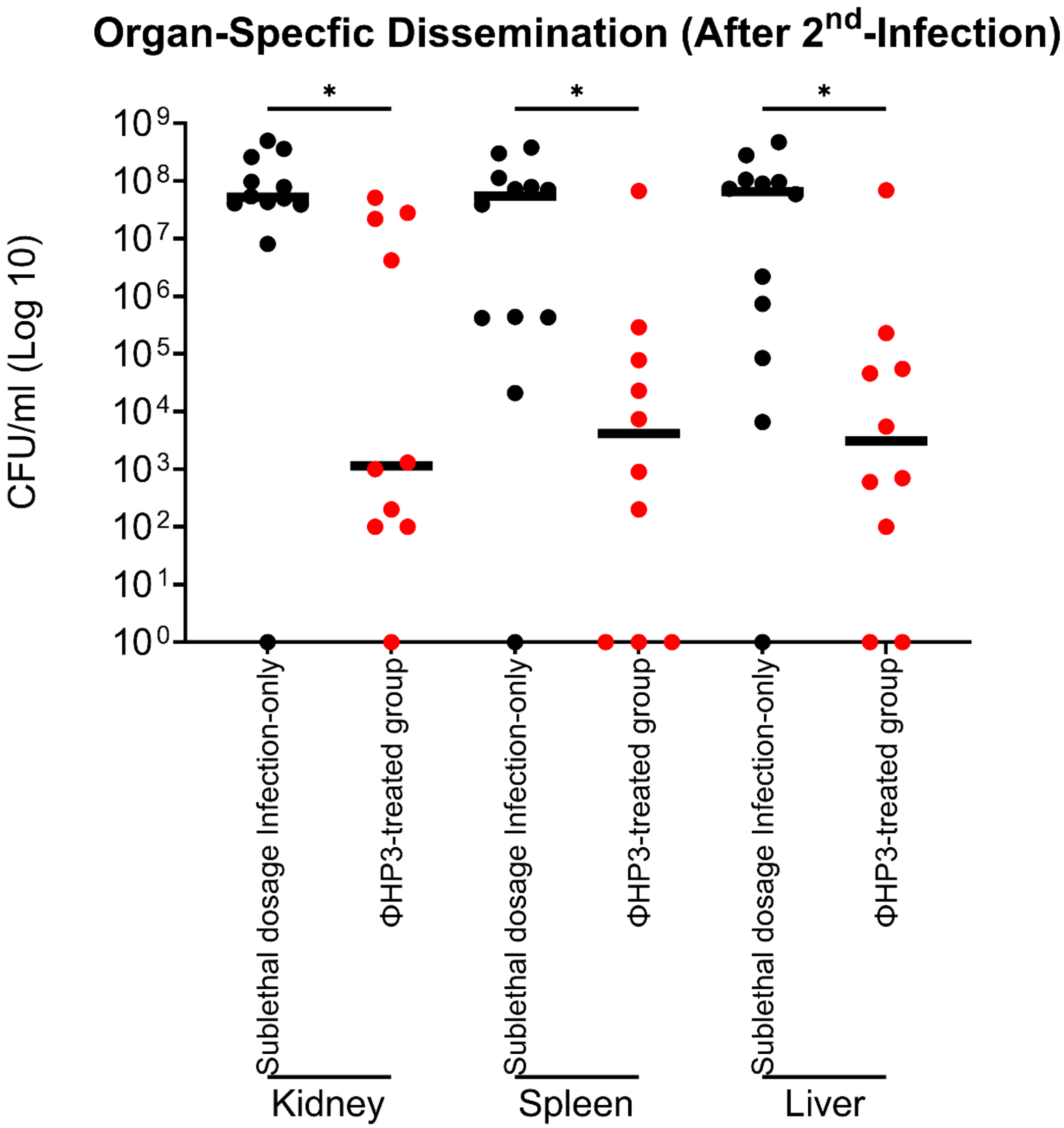

Fig. S4. Organ-specific analysis of bacterial dissemination in mice with sublethal infection.

Female BALB/cJ mice were infected intraperitoneally with 1×10^6 CFU of JJ2528. ΦHP3-treated mice received 1×10^9 PFU of ΦHP3 phage per mouse, administered eight times at 12-hour intervals. Four weeks post-treatment, mice were re-challenged with 1×10^8 CFU of JJ2528. Two weeks following re-challenge, mice were euthanized, and liver, kidneys, and spleen were homogenized and plated to measure bacterial burden. Median CFU/mL of JJ2528 by organ following 2nd infection following 2nd infection.

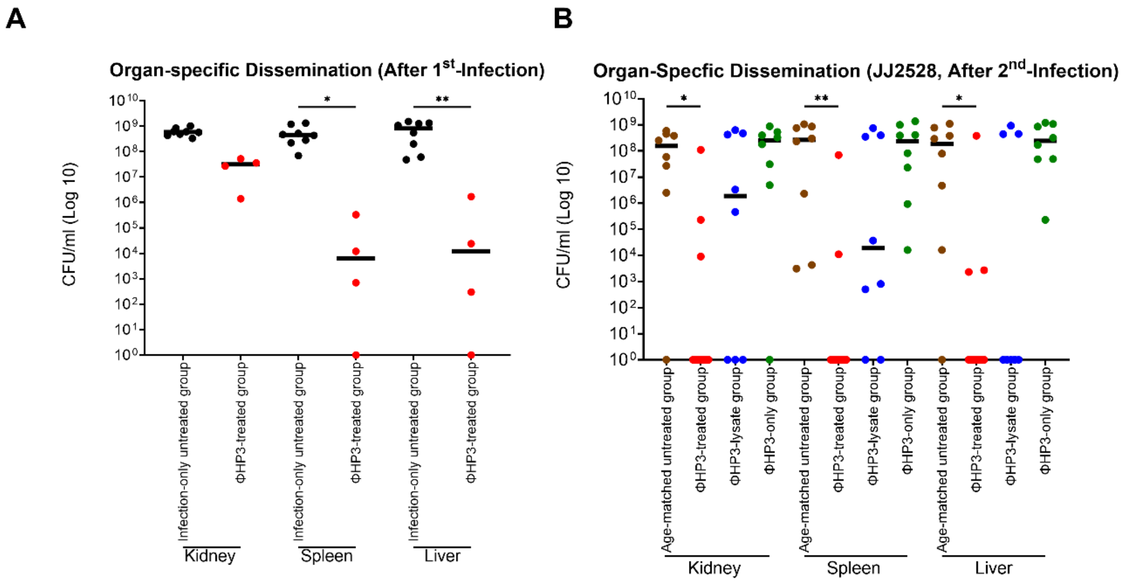

Fig. S5. Organ-specific analysis of bacterial dissemination in mice inoculated with phage only or phage-generated bacterial lysate.

Female BALB/cJ mice were infected intraperitoneally with a 1 × 10^8 CFU of JJ2528, except for the ΦHP3-only and ΦHP3-lysate groups. ΦHP3-treated and ΦHP3-only mice received either 1 × 10^9 PFU or 3 × 10^9 PFU of ΦHP3 phage per mouse, respectively, administered eight times at 12-hour intervals. ΦHP3-lysate mice received 50 µL of ΦHP3-lysate (See Methods) per mouse administered eight times at 12-hour intervals. Four weeks post-treatment, all mice were challenged with 1 × 10^8 CFU of JJ2528. Two weeks following challenge, mice were euthanized, and liver, kidneys, and spleen were homogenized and plated to measure bacterial burden. **(A)** Median CFU/mL of JJ2528 by organ following 1st and **(B)** 2nd infection.

A

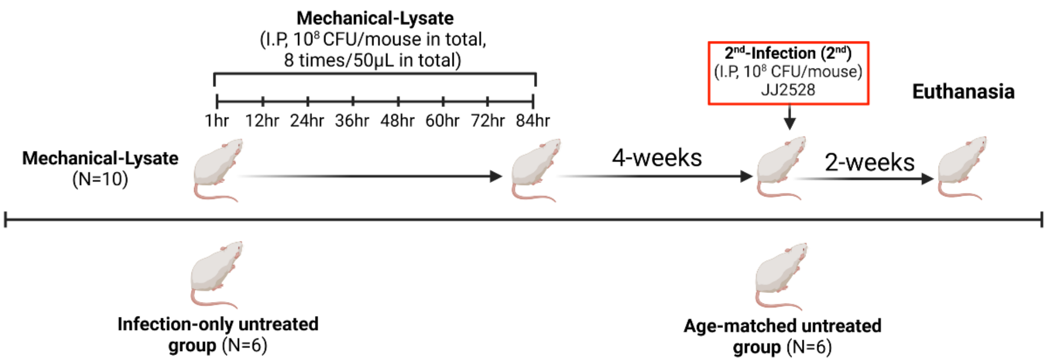

B

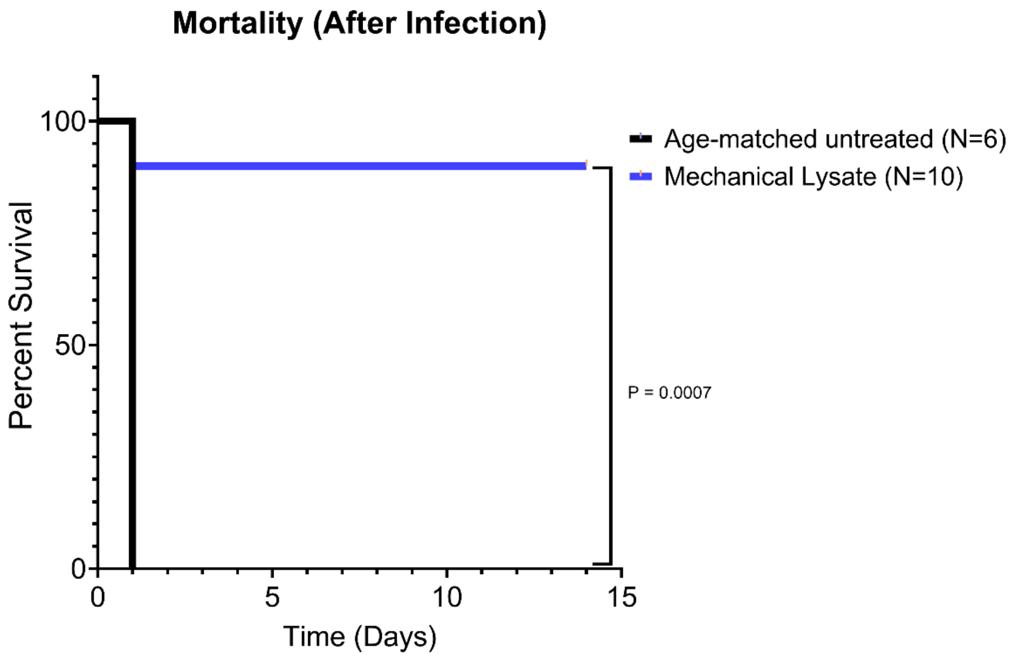

Fig. S6. Evaluation of the protective efficacy of ExPEC mechanical lysate against lethal ExPEC infection in the murine model of bacteremia

(A) Schematic of the immunization schedule using an ExPEC mechanical lysate (created with BioRender.com). Female BALB/cJ mice were inoculated intraperitoneally with 50 μL of mechanical lysate of JJ2528 (See Methods) per mouse administered eight times at 12-hour intervals. Four weeks post-treatment, all mice were challenged with 1 × 10^8 CFU of JJ2528. Two weeks following challenge, mice were euthanized, and liver, kidneys, and spleen were homogenized and plated to measure bacterial burden. The same infection-only untreated and

age-matched untreated mice were used in Figure S2 **(B)** Survival rates following the infection were analyzed using the Log-rank (Mantel-Cox) test.

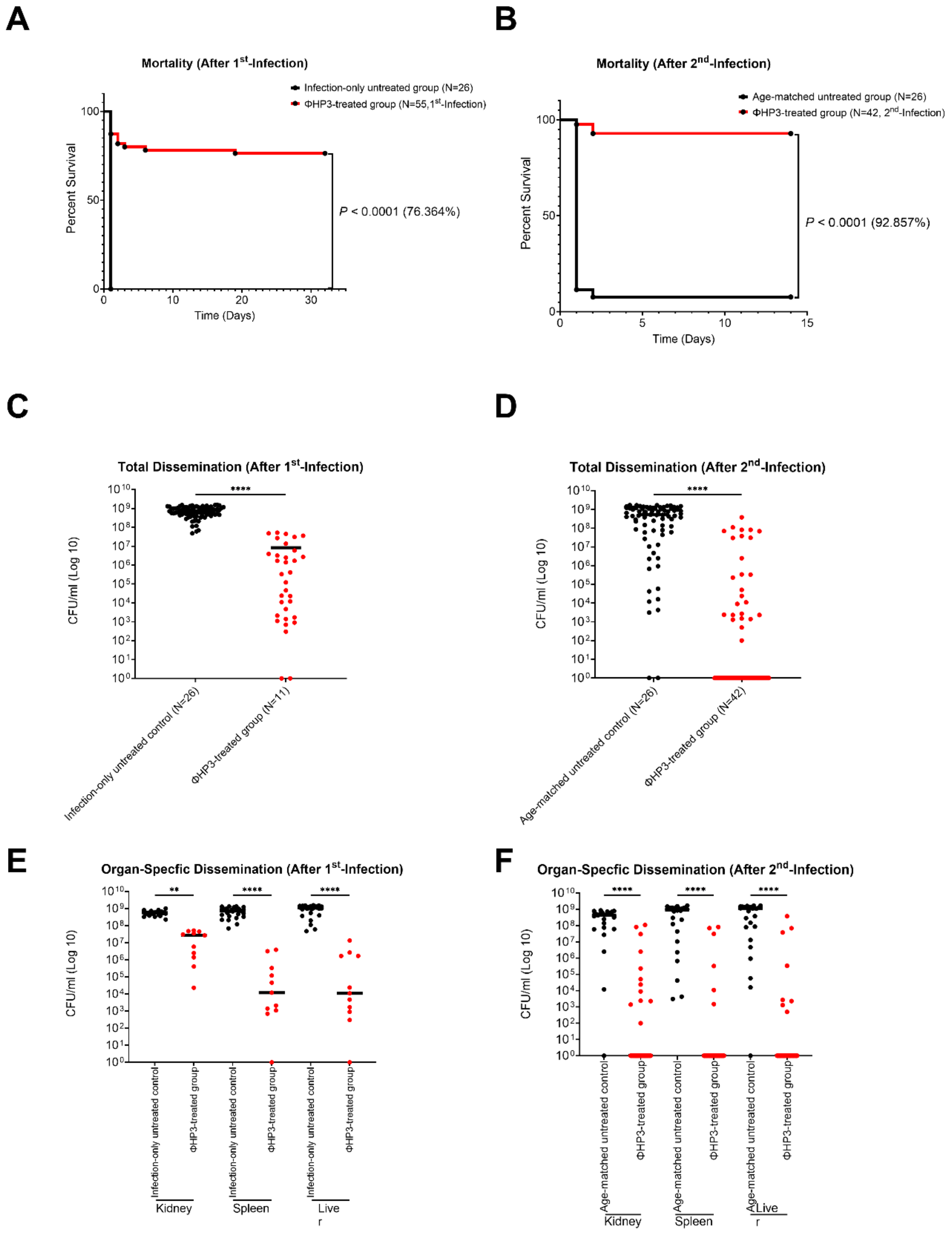

Fig. S7. Combined Analysis of Survival Rates and Bacterial Dissemination in Mice Post 1st- and 2nd- Infections Following ΦHP3 Treatment

Throughout the experiment, we used a substantial number of mice, divided into untreated control groups for both the 1st and 2nd infections, and a ΦHP3-treated group. Female BALB/cJ mice were infected intraperitoneally with a 1×10^8 CFU of JJ2528. ΦHP3-treated mice received 1×10^9 PFU of ΦHP3 phage per mouse, administered eight times at 12-hour intervals. Four weeks post-

treatment, mice were re-challenged with 1 × 10^8 CFU of JJ2528. Considering the experimental conditions described were identical across all groups, we combined and re-analyzed data from the infection-only untreated group, the age-matched untreated group, and the ΦHP3-treated group as described above to evaluate both the treatment efficacy of ΦHP3 and the protective efficacy following the 2nd-infection. In summary, after combining data under identical conditions, the cohort sizes were as follows: N = 26 for the infection-only untreated control group, N = 55 for the initially infected and ΦHP3-treated group, N = 26 for the age-matched untreated control group, and N = 42 (13 mice died following the initial infection) for the ΦHP3-treated group that survived the initial infection and were subsequently re-infected. **(A)** Survival rates following the 1st infection and **(D)** 2nd infection were analyzed using the Log-rank (Mantel-Cox) test. **(B)** Median CFU/mL of JJ2528 after 1st and **(E)** 2nd infection, combining counts from all organs. **(C)** Median CFU/mL of JJ2528 by organ following 1st and **(F)** 2nd infection.
